# Is Seeing Believing? A Practitioner’s Perspective on High-Dimensional Statistical Inference in Cancer Genomics Studies

**DOI:** 10.3390/e26090794

**Published:** 2024-09-16

**Authors:** Kun Fan, Srijana Subedi, Gongshun Yang, Xi Lu, Jie Ren, Cen Wu

**Affiliations:** 1 Department of Statistics, Kansas State University, Manhattan, KS 66506, USA; 2 Department of Pharmaceutical Health Outcomes and Policy, College of Pharmacy, University of Houston, Houston, TX 77204, USA; 3 Department of Biostatistics and Health Data Sciences, Indiana University School of Medicine, Indianapolis, IN 46202, USA

**Keywords:** exact sparsity, frequentist and Bayesian variable selection, regularized variable selection, robust Bayesian inference, uncertainty quantification

## Abstract

Variable selection methods have been extensively developed for and applied to cancer genomics data to identify important omics features associated with complex disease traits, including cancer outcomes. However, the reliability and reproducibility of the findings are in question if valid inferential procedures are not available to quantify the uncertainty of the findings. In this article, we provide a gentle but systematic review of high-dimensional frequentist and Bayesian inferential tools under sparse models which can yield uncertainty quantification measures, including confidence (or Bayesian credible) intervals, *p* values and false discovery rates (FDR). Connections in high-dimensional inferences between the two realms have been fully exploited under the “unpenalized loss function + penalty term” formulation for regularization methods and the “likelihood function × shrinkage prior” framework for regularized Bayesian analysis. In particular, we advocate for robust Bayesian variable selection in cancer genomics studies due to its ability to accommodate disease heterogeneity in the form of heavy-tailed errors and structured sparsity while providing valid statistical inference. The numerical results show that robust Bayesian analysis incorporating exact sparsity has yielded not only superior estimation and identification results but also valid Bayesian credible intervals under nominal coverage probabilities compared with alternative methods, especially in the presence of heavy-tailed model errors and outliers.

## 1. Introduction

Identifying important omics features associated with complex disease traits has remained a daunting task over the past two decades, partially due to the high-dimensional and noisy nature of omics measurements and the heterogeneity of phenotypic traits. A plethora of variable selection methods from both frequentist and Bayesian frameworks have been proposed to tackle these challenges [1,2,3]. In practice, pinpointing an important omics feature associated with a disease trait from the regression model has usually been conducted by examining whether the corresponding shrinkage estimate of the regression coefficient is exactly zero (i.e., exact sparsity) [4]. If it is nonzero, then the feature is deemed to be associated with phenotypic responses. Otherwise, the omics feature should be discarded from the final model representing the omics signature under the disease trait. Therefore, it is crucial to determine the optimal tuning parameter(s) which can impose an appropriate amount of penalties on the regression coefficients to shrink those with respect to redundant features to exactly zero [5].

For frequentist regularization methods, selections of tuning parameters have been widely investigated in the literature, including popular criteria such as the information criteria and cross-validation, which are essentially based on prediction [5]. Although these published studies have significantly improved model performance and further facilitated great success in their applications to high-throughput omics studies, concerns have been raised about the reliability of the selected features. Specifically, detecting important findings with biological implication depends on regularized estimation, while corresponding tuning parameters are chosen in line with prediction performance. However, parameter estimation and prediction are two related but distinct tasks in the statistical context. The tuning parameter which leads to the best performance in predictive modeling may not yield features truly associated with a disease phenotype. In particular, prediction conducted in a data-driven manner, such as cross-validation, has a “local” nature, meaning that we assess prediction performance based on the data at hand. But for regularized estimation, although it also shares such a local nature because we obtain shrinkage estimates with the data provided to us, we further aim to answer questions on a global scale. To what degree can we trust that the regularized coefficient is indeed not zero at the population level so that we are confident the associations have not been reported by chance using the current set of omics data? The question of whether “seeing is believing” essentially relates to the validity of the variable selection procedures used to assess the statistical significance of findings. Only by addressing this question can we appropriately handle the tension between prediction and inference in cancer genomics studies [6].

The complexity of omics studies has further jeopardized the validity of findings obtained through variable selection methods without statistical inferential tools. A well-acknowledged example is graphical LASSO, which can detect sparse graphical structures among omics features through incorporating graphical information in regularized estimation [7,8]. The sparsity of the resulting graph is determined by the tuning parameter selected in a data-driven manner, such as cross-validation. In general, embedding complicated underlying structures among omics features further increases the instability of regularization and causes irreproducibility issues. Meinshausen and Bühlmann (2010) [9] has shown that when applying graphical LASSO to a moderately large dataset with 160 genes, even a small perturbation in the tuning parameter to the order of 0.01 leads to dramatic changes in the identified graphical structure. Therefore, if data-driven procedures such as cross-validation are adopted to select the best tuning parameter, then it is unlikely to obtain the same λ (or even close ones) due to random reshuffling of the data in the procedure, which eventually leads to the irreproducibility of sparse graphical models. One way to temporarily bypass such an obstacle, as suggested in the original paper [7], is to present multiple graphs along the graphical LASSO solution path. Practitioners are strongly urged to stay vigilant when applying graphical LASSO to omics data, since sticking to one “optimal” graph and its associated omics features for downstream analysis may compromise the reproducibility of the entire study, even if variable selection is only claimed as a small component of the analysis pipeline.

Empowering variable selection methods with inferential tools has provided a potential solution to at least partially address the aforementioned validity issues. Although uncertainty quantification is not a panacea to guarantee reproducibility, statistical practice without proper inferential procedures has been acknowledged as a contributing factor to scientific irreplicability [10,11,12]. Therefore, Meinshausen and Bühlmann (2010) [9] has proposed a stability selection procedure to fit (graphical) LASSO repeatedly on subsampled data and select more stable predictors with a large selection frequency. The advantage of stability selection is twofold. First, it theoretically controls false discoveries in terms of the expected number of false selections of edges in graphical LASSO. Second, empirically, it stabilizes the sparse graphical structure along the solution path, and thus a small variation in the tuning parameter no longer results in abrupt changes in the selected graphical models. In other words, graphical LASSO is much less sensitive to tunings and thus leads to more reproducible findings. In addition, Bayesian methods, including Bayesian graphical LASSO, can also be considered to generate uncertainty measures for the identified graphs [13].

Nevertheless, uncertainty quantification measures, including the confidence intervals, *p* values, and false discovery rates (FDRs) [14,15], are not commonly reported as standard output in cancer genomics studies which utilize frequentist variable selection methods. This may be due to the theoretical challenges practitioners face in correctly understanding and applying inferential tools for frequentist sparse models. In addition, the development of high-dimensional inference methods has still been mainly focused on models with linear structures under Gaussian error assumptions, which do not account for two defining characteristics of cancer genomics studies: first, the heavy-tailed errors and outliers in phenotypic traits or omics features caused by cancer heterogeneity, and second, the structured sparsity due to the underlying patterns among omics features such as gene expressions, copy number variations and SNPs. Although frequentist robust variable selection methods have been extensively developed to address these issues [16], few of them under standard model assumptions can yield valid inference results.

On the other hand, robust Bayesian variable selection can fill gaps and complement frequentist penalization methods by providing tailored statistical inferences for cancer genomics data. For example, regularized quantile varying coefficient (VC) models are the cornerstone models for nonlinear gene–environment interaction studies under heterogeneous responses [17]. While theoretical frequentist investigations have not established the asymptotic normality of sparse VC estimators or the corresponding inference procedures in high dimensions [18,19], robust Bayesian analysis has enabled exact inference even when the sample size is not large and demonstrated satisfactory empirical coverage probabilities of Bayesian credible intervals on sparse varying coefficients [20].

In the literature, inferences with robust analysis has been mostly ignored, as indicated in Table 1, which summarizes a selected list of reviews and opinion papers on high-dimensional inferences. It shows that none of these studies have examined statistical inferences under robust regression models or hypothesis tests, regardless of whether they are frequentist [10,21,22,23,24,25,26,27,28,29,30] or Bayesian [3,31,32,33,34]. Besides, it reveals that a sheer amount and diversity of research has been generated for statistical inferences in frequentist high-dimensional analysis. This is partially because estimation and inference are decoupled under frequentist sparse models, and uncertainty quantification does not come “automatically" with estimation. On the contrary, even standard fully Bayesian analysis can utilize posterior samples drawn from MCMC to conduct high-dimensional inference and parameter estimation simultaneously [3,31,32,33,34]. Furthermore, Table 1 also suggests a lack of cross-comparison between frequentist and Bayesian methods in statistical inference, as most studies focus exclusively on one of the two. Lu and Lou (2022) [31] appears to be the only study which numerically evaluated both frequentist and Bayesian inferences. However, its interval coverage probability is for prediction instead of sparse regression coefficients as in [21,24] and our study. We refer the audience to predictive inference, especially conformal inferences, to [35,36,37].

In this review, to promote sparse robust Bayesian analysis of cancer genomics data, we conduct a small-scale but systematic survey between high-dimensional frequentist and Bayesian inference methods from the practitioner’s viewpoint. Our survey follows the well-recognized “unpenalized loss function + penalty terms” formulation for regularization methods [16,38] and “likelihood function × shrinkage prior" framework for regularized Bayesian models that root in the Bayes theorem [39,40]. Table 1 shows that most existing reviews have overlooked robust models by focusing on the least square loss and normal likelihood. Other options for unpenalized loss and likelihood have rarely been discussed. In contrast, our study investigates robust loss and likelihood, as well as their interconnections. We fully exploit connections between the frequentist and Bayesian realms by contrasting the two formulations and discussing inferences under robust sparse models. Furthermore, we evaluate the performance of multiple frequentist and Bayesian variable selection methods in terms of estimation, identification and uncertainty quantification in the presence of both normal and heavy-tailed errors in the response variables. Numerical studies have justified the advantage of robust Bayesian analysis under baseline sparse linear models, particularly highlighting its accuracy in uncertainty quantification with heterogeneous data.

## 2. Materials and Methods

We review published studies on frequentist and Bayesian high-dimensional inferences, beginning with estimation and inference in the context of standard linear regression. Consider the following linear regression model:

(1) Y=Xβ+ϵ,

where Y is an n×1 vector denoting the phenotypic trait and $X=(X_{1},...,X_{p})$ is an n×p design matrix representing the omics features with the corresponding regression coefficient vector β. Let the random error $\epsilon ={\left(\epsilon_{1},...,\epsilon_{n}\right)}^{⊤}$ with $\epsilon_{i}\overset{ind}{∼}N\left(0,\sigma^{2}\right)$, where i=1,...,n. Then, ϵ follows the multivariate normal distribution $N_{n}(0$,$\sigma^{2}I_{n})$ with an unknown variance parameter $\sigma^{2}$.

In low-dimensional settings where the sample size *n* is larger than the dimension *p*, the ordinary least square (OLS) estimator of β can be derived as $\hat{\beta }={\left(X^{⊤}X\right)}^{-1}X^{⊤}Y$ by minimizing the least square loss $||Y-{X\beta ||}^{2}$ with respect to β. This minimization procedure does not involve any distributional assumptions on random errors. Therefore, we can claim the above form of the least square estimator even without imposing a normality assumption. However, to quantify the uncertainty of $\hat{\beta }$, such a normality assumption is pivotal for establishing the distribution of $\hat{\beta }$ as $\hat{\beta }∼N_{p}\left(\beta ,\sigma^{2}{\left(X^{⊤}X\right)}^{-1}\right)$, which can then be adopted to compute the confidence intervals and *p* values and test the significance of $\hat{\beta }$. Besides, the maximum likelihood estimator of $\hat{\beta }$ is identical to the least square estimator under i.i.d. normal assumptions on random errors.

Alternatively, in the Bayesian framework, the model parameters β and $\sigma^{2}$ are treated as random variables instead of being assumed as fixed and unknown constants in the above frequentist model fitting procedures. The fundamental principle of Bayesian analysis is exemplified through the Bayes theorem, which states that posteriordistribution=likelihood×prior up to a normalizing constant (or model evidence) [39,40]. Therefore, to conduct Bayesian analysis and draw inferences from posterior distributions on β and $\sigma^{2}$, we set up the normal likelihood function and consider prior distributions of model parameters. One possible choice for assigning priors is the conjugate priors, where $\beta ∼N_{p}\left(u_{0},\sum_{0}\right)$ and $\sigma^{2}∼\mathrm{invGamma}\left(a_{0},b_{0}\right)$, which can facilitate fast sampling of the posterior samples on β and $\sigma^{2}$ drawn from MCMC algorithms. Subsequently, credible intervals and variability measures, such as standard errors, can be conveniently computed from the posterior samples to quantify the uncertainty of model parameters.

The paradigm of estimation and inference under the model (1) is highly sensitive to outlying Y observations and high-leverage points in the predictors X, which typically arise in cancer genomics studies due to disease heterogeneity. Even a single outlier can significantly impact the OLS estimates [41]. Robust estimation and inference procedures require loss functions which are more resistant to outliers than the least square loss. The robustness of three major types of estimates, M–, L– and R– estimates which are based on the maximum likelihood, linear combination of order statistics and rank tests/loss, respectively, have been widely examined in terms of efficiency and breakdown point [42]. Correspondingly, Bayesian analysis can be robustified by adopting likelihood functions utilizing heavy-tailed distributions [43,44] and Bayesian non-parametrics such as Dirichlet process mixture models [45,46].

High-throughput omics studies naturally correspond to the “large *p*, small *n*” scenario which has been extensively investigated with both the frequentist and Bayesian frameworks below.

### 2.1. The Frequentist High-Dimensional Inference

Estimation of high-dimensional linear regression models can be treated as optimization under the following regularized loss function:

(2) $$\min_{\beta }\left\{L(\beta ;Y,X)+\mathrm{Pen}(\beta ;\lambda )\right\},$$

where L(β;Y,X) represents a loss function measuring the prediction error, such as the least square loss or quantile check loss [16], Pen(β;λ) denotes the penalty term and λ is the tuning parameter which controls the bias-variance trade-off [47]. By incorporating the regularizer Pen(β;λ) into the loss function, regularized variable selection imposes constraints on the model coefficients during fitting to effectively reduce model complexity and mitigate the risk of overfitting.

In omics studies, least absolute shrinkage and selection operator (LASSO) [48] is among one of the most popular applied penalized variable selection methods with a least square loss and L1 penalty. Other baseline regularization models include SCAD [22], MCP [49], adaptive LASSO [50] and scaled LASSO [51], among others [38]. In terms of analyzing the omics data, these methods assume independence among the features and thus ignore the underlying structures among the omics measurements, which leads to the development of penalization methods accommodating structured sparsity in the forms of interrelatedness [52,53,54], groups [55,56,57], networks [58,59,60] and graphs [7,8], among others. Besides, heterogeneity is a hallmark characteristic of complex diseases such as cancer. Data contamination and outlying observations are widely observed as larger cancer subtypes are contaminated by smaller ones [16,61], which demands robust variable selection [16]. Accordingly, the unpenalized loss L(·) in formulation (2) is replaced by a robust loss such as quantile check loss, rank-based loss among others [16].

#### 2.1.1. Classical Large Sample Properties

The multivariate normality of the least square estimator (LSE) of $\hat{\beta }$ in the low-dimensional linear regression model (1) does not directly carry over to the high-dimensional penalization model (2). Therefore, developing the “oracle property” of the regularized estimator has been pivotal for high-dimensional statistical inference. Under the sparsity assumption, the true parameter $\beta^{★}$ can be expressed as $\beta^{★}={\left(\beta_{1}^{★},...,\beta_{d}^{★},\beta_{d+1}^{★},...,\beta_{p}^{★}\right)}^{⊤}={\left(\beta_{1}^{★}⊤,\beta_{2}^{★}⊤\right)}^{⊤}$, where $\beta_{1}^{★}={\left(\beta_{1}^{★},...,\beta_{d}^{★}\right)}^{⊤}$ and $\beta_{2}^{★}={\left(\beta_{d+1}^{★},...,\beta_{p}^{★}\right)}^{⊤}$ correspond to the nonzero and zero components of $\beta^{★}$, respectively (1<d<p). Ideally, we shall keep all *d* predictors with respect to $\beta_{1}^{★}$ and discard those corresponding to $\beta_{2}^{★}$. But such information is not known a priori and needs to be retrieved from the sparse estimator $\hat{\beta }={\left({\hat{\beta }}_{1},...,{\hat{\beta }}_{p}\right)}^{⊤}={\left({\hat{\beta }}_{1}^{⊤},{\hat{\beta }}_{2}^{⊤}\right)}^{⊤}$ estimated from model (2). In very rough terms, an oracle estimator $\hat{\beta }$ possesses the consistency and asymptotic normality. The consistency in variable selection, or ${\hat{\beta }}_{2}$ = 0 with a probability trending to 1, means that the zero coefficients can be correctly identified with a probability approaching 1 based on the penalized estimator $\hat{\beta }$. Such a property is usually accompanied by a rate of convergence for estimating the nonzero coefficients, such as $\left|\right|{\hat{\beta }}_{1}-\beta_{1}^{★}\left|\right|=O_{p}\left(n^{-1/2}\right)$. The asymptotic normality of ${\hat{\beta }}_{1}$ can be established as follows:

(3) $$\sqrt{n}\left({\hat{\beta }}_{1}-\beta_{1}^{★}\right)\overset{d}{\to }N_{d}\left(0_{d},\sum_{d\times d}^{★}\right),$$

where $\sum_{d\times d}^{★}$ is the covariance matrix corresponding to the true subset model. Then, the results in (3) can be adopted to compute asymptotically valid confidence intervals, *p* values and standard errors for nonzero penalized estimates to quantify the uncertainty.

However, the caveat is that the oracle property holds in the “large sample" scenario (i.e., when the sample size *n* approaches infinity). Whether it can yield exactly valid confidence intervals (with correct coverage probabilities) or *p* values on finite samples requires further examination. Baseline regularization approaches with oracle properties include SCAD, MCP and adaptive LASSO. Although LASSO is not an oracle procedure [50,62], it can still be adopted to conduct statistical inference within the selective inference framework, as we will discuss next.

#### 2.1.2. Post-Selection Inference

Post-selection inference generally refers to any approaches which can enable valid inference after data exploration [24]. By following the brief summary of the related work in Section 1.3 of Rinaldo et al. (2019) [63], we can broadly categorize these works into three major areas. First, inference with finite sample confidence intervals and *p* values calculated based on selected (or screened) models to avoid reliance on asymptotic results. For example, use LASSO (or other screening methods such as forward selection) to perform variable selection, and then conduct valid inference conditional on selected models [64,65,66,67]. The confidence intervals and *p* values are computed for each feature which enters the LASSO solution path. Second, the uniform (or simultaneous) inference methods which establish valid inferential procedures by taking every possible model selection into account and optimizing across them all, regardless of the specific model chosen [68,69,70]. The uniform inference allows for infinite revisions of selection and ensures validity even if multiple models are reported [24]. Third, inference utilizing a specific de-biased or de-sparsified penalization model which does not rely on the assumption that the nonzero components of β should not be too close to zero (i.e., the beta-min assumption), such as de-biased LASSO [71] among many others [72,73,74]. The asymptotic normality of de-biased LASSO can be established as follows [71,75]:

(4) $$\sqrt{n}\left(\hat{\beta }-\beta^{★}\right)\overset{d}{\to }N_{p}\left(0_{p},\sum_{p\times p}^{★}\right),$$

where the covariance matrix $\sum_{p\times p}^{★}=\sigma^{2}SX^{⊤}XS^{⊤}/n$ and *S* is a certain surrogate inverse matrix of the sample covariance. One distinct difference between the asymptotic normality in (3) and (4) is that the asymptotic distribution (4) holds for any $\beta^{★}$ regardless of being nonzero or extremely close to zero, while (3) only applies to the nonzero components in $\beta^{★}$. Recently, de-biased LASSO has been extended to conduct variable selection with FDR control [76,77].

#### 2.1.3. Data Shuffling via Sample Splitting and Bootstrapping

Sample splitting methods split an original dataset into training data and testing data, usually with equal sizes $\frac{n}{2}$. Model fitting using variable selection methods, such as LASSO, is performed on the training data. Then, given the selected subset of predictors whose dimension is much less than the sample size *n*, standard linear regression model can be fitted on the testing data to obtain the *p* values for the selected features. A *p* value of 1 is assigned to the unselected features. These raw *p* values for all *p* features are then adjusted for multiple testing to control the family-wise error rate [78].

The sample splitting originally proposed by Wasserman and Roeder (2009) is based on a single split [78], which is sensitive to how the entire sample is divided and may lead to unstable *p* values. Meinshausen, Meier and Bühlmann (2009) [79] has developed multi-sample splitting by performing sample splitting multiple (let us say B) times with a large B value. Then, the B *p* values for each feature are aggregated into one *p* value and adjusted for multiple testing. Variants include stability selection [9] and variable selection that controls FDR with data splitting [9,80,81,82].

In low-dimensional settings where the number of features is fixed, bootstrapping is a canonical resampling method for estimating the sampling distribution of a statistic and obtaining uncertainty measures. A high-dimensional central limit theorem (CLT) is crucial to constructing valid high-dimensional bootstrap methods. We refer readers to [25] for reviews of the theoretical results of high-dimensional CLT and relevant bootstrap methods which can generate valid confidence intervals and *p* values in high-dimensional scenarios.

#### 2.1.4. The Knock-Off Procedures

The inferential results to quantify the uncertainty of regression coefficients from high-dimensional models utilizing the methods from Section 2.1.1, Section 2.1.2, and Section 2.1.3 are in terms of the confidence intervals, *p* values and false discovery rates (FDRs), which are generally built on asymptotic (large sample) properties. In contrast, the knock-off methods select variables by exactly controlling the FDR on finite samples [83,84]. The main idea is to first construct a set of “knock-off” variables, $\tilde{X}=\left({\tilde{X}}_{1},...,{\tilde{X}}_{p}\right)$, which are not associated with the response variable Y but have a mirrored structure with the original predictors $X=(X_{1},...,X_{p})$ under certain conditions. Then, construct the filter statistic $W_{j}=w_{j}\left(\left(X,\tilde{X}\right),Y\right)$ for each feature (j=1,...,p), where the function $w_{j}$ has a flipped-sign property. Through this construction, the knock-off variables are used as negative controls, and thus a larger filter statistic, say $W_{j}$, indicates that a feature $X_{j}$ has much stronger associations with the phenotypic trait y than its knock-off counterpart $\tilde{X_{j}}$. Lastly, a threshold for $W_{j}$ is computed to allow for variable selection with exact control of the FDR on finite samples [83,84]. The knock-off methods achieve FDR control without requiring valid *p* values for individual features.

Adopting knock-offs as negative controls for variable selection has been first developed in a linear regression model with a fixed design matrix [84] and extended to high-dimensional settings [85]. The Model-X knock off [83] has relaxed [84] to allow the predictors X to be random with a known distribution and can achieve exact FDR control under nonlinear models. Although the Model-X knock-off can be adopted without knowing the true underlying relationship between the response and predictors, it relies on having exact knowledge of the distribution of X. The Model-X knock-off is robust when the distribution of X is estimated with errors [86]. In particular, good knock-off filters can be constructed based on deep generative models [87,88], sequential MCMC [89], hidden Markov models [90] and under relaxed assumptions where the distribution of X is known up to a parametric model [91]. Besides, successful applications of knock-off filters in genomics studies can be found in [92,93,94].

#### 2.1.5. Remarks on Model Assumptions, Robustness and Inference Measures

In the theoretical high-dimensional statistics literature, the word “robust” or “robustness” has been frequently encountered as we observe violations of one or more of the following assumptions: (1) the linear model assumption under model (1); (2) sparsity on the regression coefficient vector β (i.e., most of the components on β being zero); (3) the beta-min assumption on true signals, or the minimum magnitude of nonzero β components being larger than a cutoff; (4) the model error ϵ being Gaussian or having constant variance; and (5) certain conditions on the design matrix X being fixed/random, incoherent, incompatible or having restricted eigenvalues. Meanwhile, in cancer genomics studies, robustness refers to data contamination, such as outliers and heavy-tailed errors, in cancer outcomes or omics features, as well as model mis-specifications [16]. In this study, we focus on robustness in heavy-tailed errors and outliers in the response variable.

As indicated by Table 1, inferences for regularization methods have been mostly developed for non-robust models with a penalized least square loss. Published studies [95,96,97,98] are among the very few ones that have established asymptotic normality of a robust regularized estimator, all of which can be categorized under Section 2.1.1. None of these studies have assessed the empirical coverage probability of corresponding confidence intervals. One possible reason is that asymptotic properties have been established under large samples as n→∞, and many of the aforementioned assumptions (e.g., the beta-min assumption) do not necessarily apply or cannot be verified on finite samples. Besides, post-selection inference procedures have been established for high-dimensional quantile and Huber regressions [99,100,101]. Recently, He et al. (2023) [102] has developed valid inference procedures for quantile regression on large-scale data with an “increasing dimension” regime, where the sample size *n* is much larger than *p*. Our limited search suggests that, by far, finite sample evaluations on the validity of inference for robust regularization models have been only conducted in [101] in the frequentist framework, and on penalized quantile varying coefficient models in the Bayesian framework [20].

In Section 2.1.1, Section 2.1.2, Section 2.1.3, and Section 2.1.4, we provide a thorough categorization of the published frequentist studies on high-dimensional inferences, but we acknowledge that this summary may not be complete. For example, in terms of FDR, in addition to methods based on de-biasing [76,77] (Section 2.1.2), sample splitting [81,82] (Section 2.1.3) and knock-offs [83,84] (Section 2.1.4), a wide variety of FDR-guided penalization methods has also been proposed, including those based on asymptotic results [103,104], convex optimization [105] and marginal FDRs [6,106].

### 2.2. The Bayesian Inferences in High Dimensions

Bayesian analysis has provided a principled framework for delineating complex cancer genomics data, incorporating existing biological information as priors and making probabilistic inferences to better elucidate the genetic and genomic architecture of cancers, which can be concisely expressed through the following Bayesian theorem involving the high-dimensional regression vector β [39,40]:

(5) Posterior Distribution(β)∝Likelihood(β;Y,X)×Shrinkage-Prior(β),

where the shrinkage priors on the high-dimensional β is the defining characteristic that distinguishes (5) from low-dimensional Bayesian analysis. We refer readers to [3,107,108,109], among others, for comprehensive surveys on Bayesian variable selection and its application in omics studies. As the aim is to borrow strength from (robust) variable selection in frequentist framework for developing (robust) Bayesian inferences, our study will be focused on shrinkage priors that are deeply rooted in the penalized regression reviewed in Section 2.1.

#### 2.2.1. The Shrinkage Priors

**Bayesian LASSO and extensions.** We first introduce eliciting shrinkage priors through the Bayesian counterpart of frequentist regularization methods, which has been initially attempted on LASSO to develop Bayesian LASSO [48,110,111]. It has been shown that with the normal likelihood based on $N_{n}\left(Y-X\beta ,\sigma^{2}I_{n}\right)$ and an independent Laplace prior on $\beta_{j}$ (j=1,...,p): $p\left(\beta_{j}|\lambda \right)=\frac{\lambda }{2}e^{-\lambda |\beta_{j}|}$, the posterior distribution of β is proportional to $e^{-(\frac{1}{2\sigma^{2}}||Y-X\beta |{|}^{2}+\lambda \sum_{j=1}^{p}|\beta_{j}|)}$, with its maximum a posteriori (MAP) estimate being exactly the frequentist LASSO estimate. Park and Casella (2008) [110] has further refined the Laplace prior by conditioning it on $\sigma^{2}$ as follows:

(6) $$p\left(\beta |\sigma^{2}\right)=\prod_{j=1}^{p}\frac{\lambda }{2\sqrt{\sigma^{2}}}e^{-\lambda |\beta_{j}|/\sqrt{\sigma^{2}}},$$

which ensures a unimodal posterior distribution and leads to efficient sampling of the Gibbs sampler for the fully Bayesian analysis. It is worth noting that Bayesian LASSO does not lead to sparse estimates which are exactly zero. Therefore, it is often not regarded as a variable selection method. Nevertheless, as suggested in [110], a major significance of developing the Bayesian version of LASSO is to guide variable selection through interval estimates in terms of Bayesian credible intervals. The posterior samples on β can be readily generated from MCMC, which can then be utilized to construct Bayesian credible intervals, standard errors and even the entire (posterior) distribution on any $\beta_{j}$ (j=1,...,p) to quantify the uncertainty of LASSO estimates based on posterior means or medians [110]. With the interval estimates on $\beta_{j}$, variable selection can be facilitated by checking whether the credible interval of $\beta_{j}$ contains zero or not.

Bayesian LASSO assumes independence among the predictors. Therefore, it is not tailored to analyzing cancer genomics features that usually demand variable selection methods with structured sparsity to accommodate the underlying patterns within and across different types of omics features [2,61]. Instead, we can adopt other members from the LASSO family to achieve such an analysis goal, which includes elastic net [52], fused (or generalized) LASSO [53,54], group LASSO [55], sparse group LASSO [112] and graphical LASSO [7]. However, in general, it presents a greater challenge to developing inference procedures for regularization methods with complicated penalty functions under model (2), and the inferential tools are not necessarily available for these frequentist methods. On the other hand, developing the Bayesian cousins of these frequentist penalization methods has enabled statistical inference for the regression coefficients β and significantly facilitated the identification of reproducible findings. For instance, Bayesian graphical LASSO [13] can quantify the uncertainty of a graphical structure which cannot be stably detected by graphical LASSO [7,9]. Elicitation of the sparsity priors corresponding to regularization models, such as those shown in Bayesian LASSO, have been categorized as adaptive shrinkage and reviewed in detail in [3,109].

**Spike-and-slab priors.** In the literature, the development of Bayesian variable selection has initially followed a separate course from adaptive shrinkage and mainly been built upon indicator model selection, stochastic search variable selection and the model space approach [3] by leveraging the strength from sparse priors, including the spike-and-slab priors [113,114] and horseshoe priors [34]. The earliest spike-and-slab prior has the form of a “point–mass” distribution [113]:

(7) $$p\left(\beta_{j}|\gamma_{j},\sigma^{2}\right)=\left(1-\gamma_{j}\right)\delta_{0}\left(\beta_{j}\right)+\gamma_{j}p\left(\beta_{j}|\sigma^{2}\right),$$

where the point mass at zero, $\delta_{0}$, models the weak and negligible effects as the spike component and the diffused slab distribution $p(\beta_{j}|\sigma^{2})$ captures large and strong signals. The binary indicator $\gamma_{j}$ has a probability density $\theta^{\gamma_{j}}{\left(1-\theta \right)}^{1-\gamma_{j}}$ with a mixing proportion θ∈(0,1), which results in an indicator vector $\gamma ={\left(\gamma_{1},...,\gamma_{p}\right)}^{⊤}$ denoting $2^{p}$ potential models. The point–mass formulation of (7) has been extended to the mixture normal distributions as follows:

(8) $$p\left(\beta_{j}|\gamma_{j},\sigma^{2}\right)=\left(1-\gamma_{j}\right)N\left(0,c_{1}^{2}\sigma^{2}\right)+\gamma_{j}N\left(0,c_{2}^{2}\sigma^{2}\right),$$

where $0<c_{1}^{2}<c_{2}^{2}$ leads to spiky and flat normal distributions. A stochastic search variable selection (SSVS) approach is proposed to examine the frequencies for all $2^{p}$ models with the posterior samples obtained from Gibbs sampling [114]. The top-ranked subset models in terms of selection frequency and corresponding predictive performance are adopted to recommend variables for selection. However, when *p* becomes large, it is no longer realistic to examine all $2^{p}$ models because of the curse of dimensionality. The median probability model has been widely adopted to determine whether a predictor should be included or discarded [115,116]. Specifically, if the posterior inclusion probability is larger than a cutoff of 0.5 (i.e., $\mathrm{Pr}(\gamma_{j}|Y,X)>0.5$, j=1,...,p), then the feature is selected.

Investigating all the $2^{p}$ submodels in variable selection essentially entails the idea of Bayesian model averaging which combines all of the candidate models to learn model parameters and account for model uncertainty [117,118]. When such a combination or averaging is not feasible due to the curse of dimensionality, adopting the median probability as a threshold to determine the selected predictors makes Bayesian variable selection possible in high-dimensional settings. By construction of the spike-and-slab priors in formulation (7), if a posterior inclusion probability $\mathrm{Pr}(\gamma_{j}|Y,X)$ is below 0.5, then the corresponding posterior median estimates on $\beta_{j}$ must be zero exactly. Pursuit of the exact sparsity has been the major theme of developing frequentist regularization methods, which has also resulted in novel Bayesian variable selection methods of the frequentist spirit. For example, Bayesian LASSO estimates using the Laplacian prior of (6) cannot yield exact 0 posterior median or mean estimates in general. However, we can consider incorporating the spike-and-slab prior in the Bayesian LASSO hierarchical model as follows:

(9) $$\begin{array}{cc} \beta_{j}|\gamma_{j},\tau_{j}^{2},\sigma^{2} & \overset{ind}{∼}\left(1-\gamma_{j}\right)\delta_{0}\left(\beta_{j}\right)+\gamma_{j}N\left(0,\;\sigma^{2}\tau_{j}^{2}\right),\\ \gamma_{j}|\theta & \overset{ind}{∼}\mathrm{Bernoulli}\left(\theta \right),\\ \tau_{j}^{2}|\lambda & \overset{ind}{∼}\mathrm{Gamma}\left(1,\;\frac{\lambda^{2}}{2}\right), \end{array}$$

which is exactly equivalent to formulation (6) as a scale mixture of normal distribution with the Gamma density if $\gamma_{j}=1$. The failure of Bayesian LASSO to produce exact 0 posterior estimates has not only caused large estimation errors but also inferior identification performance due to the overly wide credible intervals. When $\gamma_{j}=0$, the regression coefficient $\beta_{j}$ will be modeled as the point mass at zero, thus achieving the exact sparsity. The gains of the hierarchical model specified by formulation (9) are at least twofold. First, the exact sparsity leads to better estimation and identification performance compared with formulation (6). Thus, the accuracy of associated uncertainty quantification measures in terms of credible intervals will improve accordingly. Second, in cancer genomics studies, the hierarchical structure in (9) can be potentially extended to adaptive shrinkage based on prior elicitation procedures to account for the structured sparsity of multi-omics features, making it possible to conduct statistical inference under complicated sparse models. For instance, published studies have demonstrated that the spike-and-slab prior of (7) can be generalized to a multivariate case for the Bayesian group LASSO, which results in superior performance compared with their non-spike-and-slab counterparts [119,120], especially in terms of Bayesian inferences utilizing credible intervals [20].

**Exact sparsity, a judgment call?** Under spike-and-slab priors, questions on whether the median probability 0.5 adopted as the cutoff to determine the selected features is a judgment call have been raised. It has been argued that the Bayesian inference returns MCMC samples of the binary indicators $\gamma_{j}$, which are then averaged to obtain a posterior inclusion probability $\mathrm{Pr}(\gamma_{j}|Y,X)$ for $\beta_{j}$. Therefore, whether the cutoff should be 0.5, 0.7 or 0.9 is a judgment call. However, the median probability threshold has a particular implication under the point-mass structure of (7), which has been referred as the “gold standard” for sparse Bayesian models [121,122,123]. When $\mathrm{Pr}(\gamma_{j}|Y,X)$ is less than 0.5, the posterior median estimates of $\beta_{j}$ is zero exactly. Such an exact sparsity does not hold for other cutoff choices in general. In addition, we refer readers to theoretical investigations on the merits of the median probability model [115,116]. Although other shrinkage priors, such as the horseshoe prior based on well-established global-local regularization, can also produce a (pseudo) posterior inclusion probability with a user-defined cutoff [34,122,124,125], we should distinguish the thresholds used for the two scenarios since, in terms of both prior structure and mechanisms of conducting Bayesian shrinkage estimation, the two-group spike-and-slab prior fundamentally differs from the one-group global-local shrinkage priors of the form below:

(10) $$\begin{array}{cc} \beta_{j}|\lambda_{j},\tau^{2} & \overset{ind}{∼}N\left(0,\lambda_{j}^{2}\tau^{2}\right),\\ \lambda_{j} & \overset{ind}{∼}p\left(\lambda_{j}\right),\\ \left(\tau ,\sigma^{2}\right) & ∼p(\tau ,\sigma^{2}), \end{array}$$

where the global term τ shrinks all regression coefficients toward zero and $\lambda_{j}$ acts locally through its heavy tails so that the $\beta_{j}$’s are not penalized too much. If the local term $\lambda_{j}$ follows a half-Cauchy distribution (i.e., $C^{+}\left(0,1\right)$), then the formulation of (10) leads to the horseshoe prior.

**Spike-and-slab LASSO.** To further reduce the computational burden of conducting MCMC-based fully Bayesian inference, the spike-and-slab LASSO (SSL) prior has been proposed to borrow strength from the frequentist and Bayesian LASSO while overcoming their respective limitations [121,126,127]. Specifically, the SSL prior can be expressed as follows:

(11) $$\begin{array}{cc} \beta_{j}|\gamma_{j},s_{0},s_{1} & \overset{ind}{∼}\left(1-\gamma_{j}\right)p\left(\beta_{j}|s_{0}\right)+\gamma_{j}p\left(\beta_{j}|s_{1}\right),\\ \gamma_{j}|\theta & \overset{ind}{∼}\mathrm{Bernoulli}\left(\theta \right),\\ \theta & ∼\mathrm{Beta}(a_{0},b_{0}), \end{array}$$

where the conditional Laplace prior $p\left(\beta_{j}|s\right)=\frac{1}{2s}e^{-|\beta_{j}|/s}$ is defined as the spike distribution $p(\beta_{j}|s_{0})$ for $s=s_{0}$ and the slab distribution $p(\beta_{j}|s_{1})$ for $s=s_{1}$ with a scale parameter $s_{1}>s_{0}>0$. The SSL prior distinguishes itself from the fully Bayesian formulation (9) in that, first, it has a continuous spike-and-slab mixture double exponential distribution, and second, the scale parameters $s_{0}$ and $s_{1}$ are treated as deterministic and selected based on data-driven procedures such as cross-validation, instead of being assumed as random and assigned with hyper-priors in fully Bayesian analysis under formulation (9). Such a difference is essential for implementing an extremely fast EM algorithm based on coordinate descent [128,129,130] or least angle regression [131] to find the posterior mode of β. Spike-and-slab LASSO enjoys selective shrinkage and self-adaptivity to the sparsity pattern utilizing a solution path algorithm. It has also been extended to models for survival and categorical outcomes, as well as phenotypes with outliers in cancer genomics studies [132,133,134]. Moreover, Nie and Ročková (2023) [135] has established that through the weighted Bayesian bootstrap [136], an approximate posterior sampling procedure can be developed for spike-and-slab LASSO to avoid the cost of MCMC-based posterior sampling under high dimensions.

**Remarks.** The surveyed sparse priors all have strong frequentist implications. For example, Mallick and Yi (2013) [109] has demonstrated details to develop a Bayesian LASSO family of priors from their corresponding frequentist regularization models. The spike-and-slab prior can be incorporated within both Bayesian LASSO as in formulation (9) and LASSO as in formulation (11) to borrow strength from frequentist regularization. Furthermore, Bhadra et al. (2019) [34] has shown how the LASSO model facilitates the development of horseshoe priors among global-local priors. In addition, other sparse priors and models have also been proposed in published studies, from indicator model selection [137,138] to the more recent Dirichlet Laplace prior [139] and horseshoe+ prior [140], as well as non-local priors [141,142].

#### 2.2.2. The Robust Likelihood

Our explorations of high-dimensional Bayesian inferences are largely focused on shrinkage priors while assuming the normal likelihood. However, the disease phenotypic traits in cancer genomics studies are frequently contaminated by outliers and have skewed distributions due to the heterogeneity of cancer. Bayesian analysis of the omics data under non-robust normal likelihood causes not only biased estimation and identification of false findings but also inaccurate uncertainty quantification measures. Therefore, there is a pressing need to adopt a robust likelihood to accommodate widely observed heavy-tailed errors in the phenotype. The asymmetric Laplace distribution (ALD) defined below for i.i.d. random errors $\epsilon_{i}$ is pivotal for formulating the widely used Bayesian quantile regression ([43,143]):

(12) $$f\left(\epsilon_{i}|\xi \right)=\tau \left(1-\tau \right)\xi \exp\left[-\xi \rho_{\tau }\left(\epsilon_{i}\right)\right]=\tau \left(1-\tau \right)\xi \left\{\begin{array}{cc} e^{-\xi \tau \epsilon_{i}}, & \mathrm{if}\;\epsilon_{i}\geq 0\\ e^{\xi \left(1-\tau \right)\epsilon_{i}}, & \mathrm{if}\;\epsilon_{i}<0, \end{array}\right.$$

where $\xi^{-1}$ is a scale parameter defining the skewness of the distribution and τ∈(0,1) is a fixed quantile level. The ALD density corresponds to $\rho_{\tau }\left(\epsilon_{i}\right)=\epsilon_{i}\left\{\tau -I\left(\epsilon_{i}<0\right)\right\}$, the check loss function in quantile regression, which has been among the most broadly employed robust loss functions in bioinformatics studies [16]. Bayesian regularized quantile regression based on ALD likelihood has been proposed in [144], and recently developed to account for structured sparsity in omics studies, including gene–environment interactions [17,145,146]. In addition to adopting the parametric ALD to specify the likelihood, Reich et al. (2010) [147] has proposed to model the error non-parametrically as an infinite mixture of normal densities, subject to stochastic constraints for Bayesian quantile regression.

Although there are other choices of robust likelihood for high-dimensional Bayesian analysis, such as the mixture of Gaussians from finite mixture models [148] and Dirichlet process mixture models [149,150,151], surveys show that the development of variable selection methods under these likelihood functions are mainly limited to baseline settings, where structured sparsity among omics features are ignored [152,153]. In fact, the major goals are to perform subgroup or clustering analysis in order to identify homogeneous subgroups or clusters from a heterogeneous population in a supervised or unsupervised manner. For the convenience of setting up Bayesian hierarchical models and deriving MCMC algorithms, Gaussian mixture assumptions are usually adopted, which are not considered as robust methods for handling outliers or heavy-tailed errors compared to the mixture regression under *t* distributions [154,155,156,157].

### 2.3. Connections between High-Dimensional Frequentist and Bayesian Methods

**Figure 1:**
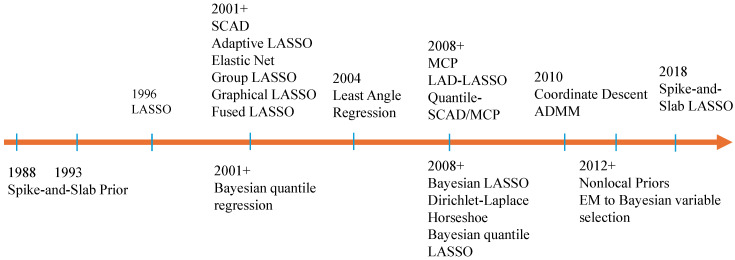
A brief history of frequentist and Bayesian variable selection methods.

We present a brief history of frequentist and Bayesian variable selection methods over the past three decades in Figure 1. A high-level connection between the two realms can be readily observed by comparing the frequentist “unpenalized loss function + penalty terms" formulation of (2) and the “likelihood × shrinkage prior" structure from the Bayesian theorem in (5). Regularization methods adopt the unpenalized loss function to measure the lack of fit and penalty functions to impose sparsity. Alternatively, Bayesian ones utilize the likelihood function to describe the plausibility of data governed by model parameters and achieve variable selection via incorporating shrinkage priors in a Bayesian hierarchical model.

The correspondence between formulations (2) and (5) can be illustrated through not only LASSO and Bayesian LASSO but also their robust cousins, among many other examples. Specifically, LAD-LASSO has the “L1 loss function + L1 penalty” structure [96,158], while Bayesian LAD-LASSO is of the form of “Laplacian likelihood × Laplace prior”, where the Laplacian likelihood is a special case of ALD at the 50% quantile level in (12). Table 1 indicates that statistical inferences for robust variable selection methods have been largely overlooked, let alone quantifying uncertainty for more complicated robust models which can accommodate structured sparsity in cancer genomics studies. Therefore, developing the Bayesian counterpart of frequentist robust methods is of particular importance in conducting valid statistical inference lacking in the frequentist methods.

**Exact sparsity: a second look.** Retrieving the literature on variable selection in the 1990s from Figure 1 reflects the philosophical differences between high-dimensional frequentist and Bayesian methods during those early days. The soft thresholding rule leads to exact sparsity of LASSO estimates under a fixed tuning parameter which should be carefully selected in a data-driven manner [4,48]. While LASSO has exhibited deterministic behavior and lacks inferential procedures, Bayesian variable selection using spike-and-slab priors proceeds with a probabilistic approach, averaging over all candidate models and quantifying their uncertainty [113,114]. The Bayesian methods do not prioritize the pursuit of exact sparsity. However, the curse of dimensionality necessitates consideration of exact sparsity when using spike-and-slab priors, particularly through median probability thresholds, which hold significant theoretical and empirical implications rather than being a judgment call [115,116]. The trend of embracing shrinkage eventually facilitates development of Bayesian methods such as Bayesian LASSO and spike-and-slab LASSO, which fully capitalize on the frequentist way of inducing shrinkage estimates [110,127].

Applying a user-defined cutoff to obtain exact sparsity is not rare and also achieves good performance in certain scenarios, such as in constrained convex optimization with the alternating direction method of multipliers (ADMM) [159] and penalized estimating equation (PGEE) with the Newton–Raphson type of model fitting algorithms [160], where exact 0 estimates cannot be yielded automatically as in the LASSO solution under the soft thresholding rule unless a user-specified threshold is enabled to shrink small fractions of nonzero estimates to zero. Nevertheless, using an ad hoc cutoff to decide the selected omics features leads to problems in models tailored for structured sparsity in cancer genomics studies. For example, when group-level selection is demanded in longitudinal studies [161,162], applying the threshold as a judgment call can easily result in a sparse group selection which violates the “group in, group out” hierarchy in group LASSO [112,161,163]. Such an issue has also been reported in [164], where a multivariate conditional Laplace prior has been adopted to develop Bayesian group LASSO in non-parametric varying coefficient models. Similar to the prior in (6), its extension to a multivariate prior cannot produce exact 0 estimates. Therefore, employing a 95% credible interval to identify features can again lead to selecting a sparse group instead of the entire group. These issues no longer exist if the proposed method can automatically yield exact group-level sparsity, regardless of being frequentist [165,166,167] or Bayesian [20,120].

**Inferences with robust sparse models.** In bioinformatics studies, the demand for robust variable selection arises due to (1) outliers and data contamination in the response variable (e.g., cancer outcomes and disease phenotypes), (2) outlying observations and contamination in the omics features (or predictor space), which has also been termed as high-leverage point in the robust literature [42], and (3) model mis-specifications, such as assuming a main effect model in the analysis of interaction studies [16]. Robustifying regularization methods against heavy-tailed distributions in the response variable requires a robust unpenalized loss under (2), such as the quantile check loss, Huber loss [42], rank-based loss [168,169] and least trimmed squares [170,171], among others [16]. Besides, robustness against a high leverage point usually depends on outlier detection and trimming of the loss functions [171,172], or adopting and developing a loss function with a high breakdown point and bounded influence functions (which quantifies the stability of regularized estimators with an infinitesimal contamination) in the predictor space [98,173]. Furthermore, handling model mis-specifications requires that the sparse model can accommodate structured sparsity among the omics features. The challenge of developing valid statistical inference procedures for robust sparse models increases exponentially compared with their non-robust counterparts. This is because many conventional model assumptions listed in Section 2.1.5, which are essential for establishing large sample properties (Section 2.1.1), are no longer applicable under robust models. The asymptotic normality of a robust regularized estimator has been established in a limited number of studies on linear (main effect) models, including [95,96,97,98]. Nevertheless, none of these studies has assessed the validity of inference measures (e.g., the coverage probability of corresponding confidence intervals). The inferences for robust high-dimensional models utilizing post-selection inference (Section 2.1.2), data shuffling (Section 2.1.3) and the knock-off procedures (Section 2.1.4) have rarely been reported.

On the contrary, robust Bayesian variable selection methods have been relatively underdeveloped. Although a robust likelihood function in (5) ensures that Bayesian methods are insensitive to long-tailed distributions and outliers in cancer outcomes (or disease traits), unlike formulating the ALD likelihood in (12) based on the quantile check loss [43,143] or the finite mixture models which can be readily adopted in both frequentist and Bayesian realms [148], for many robust losses, such as the rank-based ones and the least trimmed squares among those reviewed in [16,174], it is difficult or even infeasible to specify a corresponding likelihood function. Therefore, Bayesian counterparts based on these robust losses are not available in general. Furthermore, robustness with respect to a high leverage point in the predictor space requires well-defined concepts, including the high leverage point, breakdown point and influence functions whose Bayesian counterparts are not available in the first place. Thus, Bayesian methods robust to contamination in the predictors have seldom been examined.

Nevertheless, robust Bayesian variable selection still holds great promise since fully Bayesian analysis enables exact inference even with a limited sample size. To date, the utility of robust Bayesian inferences under baseline sparse models has not been assessed yet. Although the ALD likelihood has been predominately used in specifying the likelihood in Bayesian (regularized) quantile regression, Yang et al. (2016) [175] has pointed out that it is a working likelihood to ensure that minimization of the quantile check loss is equivalent to maximizing the corresponding likelihood under (12). Inferences under the working likelihood are not accurate in low-dimension models, and a posterior variance adjustment step has to be imposed. Besides, Reich et al. (2010) [147] has adopted the Dirichlet process prior to model residuals in low-dimensional Bayesian quantile regression and shown that it can maintain a desired coverage probability at a given frequentist nominal level. In high-dimensional settings, we will demonstrate in the simulation study that exact sparsity can facilitate Bayesian regularized quantile regression to yield honest credible interval coverage, even in the presence of outliers and long-tailed distributions.

**Approximate inference.** The computational cost of MCMC is prohibitively high for large-scale data. To facilitate fast Bayesian computation and inferences, multiple approximate procedures have been developed to bypass the sampling of an entire posterior distribution for inference. The overall strategy is to turn Bayesian estimation and inferences into a frequentist optimization problem. Spike-and-slab LASSO has provided one promising solution by first finding the posterior mode of β through EM algorithms and subsequently conducting approximate Bayesian inferences through weighted Bayesian bootstrapping [126,127,135]. Updating $\hat{\beta }$ in the M step of the EM algorithm follows the LASSO soft thresholding rule. In addition, in the machine learning community, variational Bayes methods have been widely developed for the same purpose [176]. Finding an approximate distribution that is closest to the exact posterior in terms of KL divergence can again be turned into an optimization problem based on coordinate ascent, among other optimization algorithms [177]. Recently, variational Bayes has found successful applications in genomics studies [178,179,180]. We refer readers interested in other relevant approximate Bayesian computation methods to [181,182].

## 3. Simulation Study

We have assessed the performance of six representative shrinkage methods in terms of estimation, identification and uncertainty quantification. There are four fully Bayesian methods: Bayesian LASSO (BL) [110], robust Bayesian LASSO (RBL) [144], Bayesian LASSO with spike-and-slab prior (BLSS) [146,183] and robust Bayesian LASSO with spike-and-slab prior (RBLSS) [146]. RBL and RBLSS are the corresponding Bayesian regularized quantile regressions at the 50% quantile level. All of the Bayesian methods can be implemented using the codes from R package *roben (ver 0.1.1)* [184], and derivations of their Gibbs samplers are available in [146]. In addition, the frequentist regularization methods Debiased-LASSO [71] and Selective Inference based on LASSO [65] have also been included. They have been implemented using R packages *hdi* [21] (ver 0.1.9) and *selectiveInference (ver 1.2.5)*, respectively. All of the methods are non-robust, except RBL and RBLSS. We have adopted the point-mass spike-and-slab prior for BLSS and RBLSS so that both can lead to exact 0 estimates.

The data have been generated from the sparse model described in Section 2.1.1 with a sample size *n* = 100 and dimensionality *p* = 501. The predictors X have been simulated from a multivariate normal distribution with (1) auto-regression (AR-1) covariance structure where the *i*th and *j*th predictors have a correlation $0.5^{\left|i-j\right|}$ and (2) a diagonal covariance matrix representing independence among the features. The true values of the regression coefficients in the coefficient vector are $\beta^{★}={\left(1,1.5,2,0,...,0\right)}^{⊤}$, where the first three components are nonzero (i.e., $\beta_{1}^{★}={\left(1,1.5,2\right)}^{⊤}$) and the rest of the coefficients are zero (i.e., $\beta_{2}^{★}={\left(0,...,0\right)}^{⊤}$).The intercept is set to zero. We have considered two probability distributions for the random error ϵ: a standard normal distribution N(0,1) and a heavy-tailed distribution *t*(2). The simulation study has been conducted over 1000 replicates.

For all four Bayesian methods, we have collected posterior samples from 10,000 MCMC iterations after discarding the first 5000 as burn-ins. For BLSS and RBLSS which have incorporated the spike-and-slab priors, if the binary indicator $\gamma_{j}^{\left(k\right)}$ = 1, then this shows that feature $X_{j}$ is included in the model at the *k*th MCMC iteration. Then, the posterior inclusion probability of keeping $X_{j}$ in the final model can be computed as

$$\mathrm{Pr}\left(\gamma_{j}|Y,X\right)=\frac{1}{M}\sum_{k=1}^{M}\gamma_{j}^{\left(k\right)},\;j=1,\ldots ,p.$$

where *M* is the number of posterior samples. Larger posterior inclusion probabilities indicate stronger empirical support that the *j*th predictor is associated with the response. We use the median probability 0.5 to determine whether the predictor is selected, which corresponds to the median probability model (MPM) defined in [115,116], as the model consisting of predictors with a posterior inclusion probability larger than 0.5 when the goal is to select a single, final model. For BL and RBL, two Bayesian methods without the spike-and-slab priors, the 95% credible interval is adopted to claim identified predictors if they do not cover zero. The selected features are then used to compute the number of true positives (TPs) and false positives (FPs). We have recorded the posterior medians $\hat{\beta }$, so the L1 estimation error can be calculated as $\sum_{j=1}^{p}|\beta_{j}-\hat{\beta_{j}}|$. In terms of uncertainty quantification, the credible intervals for four Bayesian methods and confidence intervals for the two frequentist methods have been evaluated given a nominal level of 95%.

Table 2 summarizes all the numerical results under AR1 correlations among the predictors. Under an error of N(0,1), we can observe that BLSS has the smallest L1 estimation error (0.305 (sd 0.146)) on the true covariates, and Debiased-LASSO produces the smallest estimation error on the non-important predictors (0.061 (sd 0.021)). BLSS identifies all of the correct signals with a small fraction of FPs (0.096 (sd 0.333)) which is slightly better than that for RBLSS. This shows that BL and RBL have yielded much larger estimation errors compared with other approaches, especially for the irrelevant covariates (10.782 (sd 0.777) under BL and 14.739 (sd 0.818) under RBL). Such a pattern is consistent with our expectation because both approaches cannot achieve exact sparsity, which naturally leads to much larger estimation errors for those zero coefficients associated with noisy features. Pursuing exact sparsity has a direct impact on the estimation performances.

Regarding statistical inference under an error of N(0,1), for BLSS, the coverage probabilities on the coefficients of the three true covariates are 0.942, 0.945 and 0.949, which are all extremely close to the 95% nominal level. The average length of the three credible intervals is among the smallest over all methods under comparison. RBLSS and Debiased-LASSO are slightly inferior in terms of their coverage probabilities for nonzero coefficients. Additionally, we have computed the average coverage probability for all the false predictors, together with a corresponding average length of the credible or confidence intervals, which indicates that Debiased-LASSO can still lead to confidence intervals with ideal coverage rates and lengths. For all of the Bayesian methods, we observe a super-efficient phenomenon for noisy features [21,75], meaning that the credible intervals have a (nearly) 100% coverage probability. This is consistent with the results reported in [21,75] for both the frequentist and Bayesian sparse models. As shown in [75], the spike-and-slab prior is highly concentrated on the 0 estimates with noisy features. Bayesian credible intervals can be quite narrow, yet they always have 100% coverage rates asymptotically.

In Table 2, the average lengths under LASSO-based Selective Inference are not reported, since many confidence intervals have an infinite upper or lower bound. Selective Inference is a conditional method which only computes the confidence intervals for the selected predictors. We have skipped those replicates (less than 5%, especially under the *t*(2) distribution) where none of the predictors have been selected. Since Selective Inference can produce confidence intervals for features identified under any fixed tuning parameter λ along the LASSO solution path, for reproducibility of the simulation study, we have fixed the tuning parameter as $\lambda_{\mathrm{opt}}$ which leads to the smallest cross-validation error.

The lower panel of Table 2 shows that the performances of all methods have decreased under a heavy-tailed t(2) error compared with those under *N*(0,1). The robust RBLSS outperforms the other models by having the smallest estimation error and the highest number of true positives (TPs) while giving almost zero false positives (FPs). The benefit of incorporating exact sparsity is evident. Although BLSS lacks robustness, its estimation errors are 1.353 (sd 0.941) for the true signals and 4.911 (sd 14.225) for the false ones, which are much lower compared with the corresponding errors of 2.033 (sd 0.451) and 23.405 (sd 4.082) observed with the robust RBL that cannot yield exact 0 posterior median estimates. In addition, the number of TPs of RBLSS is 2.948 (sd 0.222), larger than those from the others, while its number of FP is merely 0.042 (sd 0.186).

Our simulations have demonstrated the empirical performance of the inferential procedures under the heavy-tailed error. Under *t*(2), the coverage probabilities of RBLSS for the three nonzero coefficients of the true predictors are 0.954, 0.960 and 0.964, respectively, which are again close to the 95% nominal level. The average lengths of the three confidence intervals across 1000 replicates are 0.833, 0.856 and 0.728, which are larger than the corresponding lengths of 0.476, 0.531 and 0.477 under the N(0,1) model error. Debiased-LASSO has also demonstrated a stable performance in terms of the coverage rates for the coefficients of both the true and noisy features. However, it is important to note that the lengths of the confidence intervals have increased dramatically. For instance, for the three nonzero true coefficients, the average length has risen from 0.483, 0.482 and 0.481 under N(0,1) to 1.418, 1.409 and 1.412 under *t*(2), respectively. The coverage rates for the zero coefficients with false predictors for the four Bayesian approaches are close to 1. Therefore, the super-efficient phenomenon also exists under the heavy-tailed error. Meanwhile, the non-robust Debiased-LASSO has maintained a good coverage probability (i.e., 0.955) for the zero coefficient, although the average length of the confidence intervals is much larger than that from the N(0,1) case.

**Figure 2:**
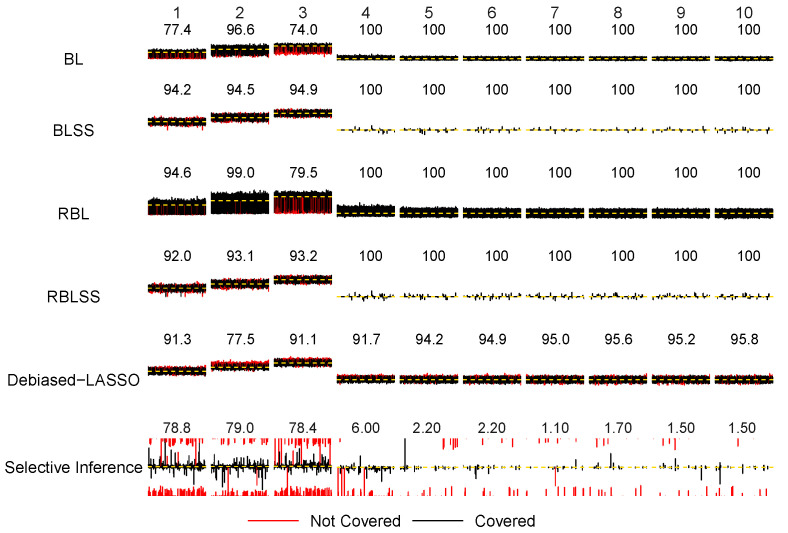
Confidence and credible intervals and their coverage probabilities under a 95% nominal level for the first 10 predictors, where the first 3 are true nonzero coefficients. Data were simulated under AR(1) correlation (n,p)=(100,501) and N(0,1) error across 1000 replicates.

**Figure 3:**
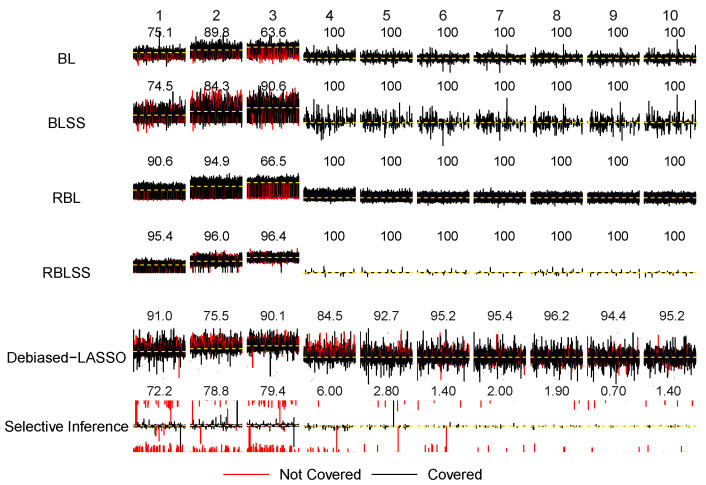
Confidence and credible intervals and their coverage probabilities under a 95% nominal level for the first 10 predictors, where the first 3 are true nonzero coefficients. Data were simulated under AR(1) correlation (n,p)=(100,501) and *t*(2) error across 1000 replicates.

The inference results for the confidence and credible intervals and their coverage probabilities in Table 2 have been visualized in Figure 2 and Figure 3 for the N(0,1) and *t*(2) errors, respectively. For convenience, we have provided plots for the first 10 predictors, where the first 3 are true nonzero coefficients and the remaining 7 are true zero coefficients. The magnitude of the coefficients is represented by the heights of the black horizontal bars. We have placed all of the intervals obtained from 1000 replicates over the horizontal bar with each interval colored either black or red. Black indicates that the interval covers the true coefficient, while red means that it does not. The coverage rates for each coefficient are displayed above the horizontal bars. The y-scale range in the plots is from −5 to 5 for all methods except Selective Inference which has generated a large number of CIs with infinite upper or lower bounds. We has thus adopted a much larger *y*-scale range on (−50,50) for the last row in the plots. The coverage probabilities have been obtained over 1000 replicates except for the LASSO-based Selective Inference, since at a certain number of replicates (less than 5%), none of the predictors are selected. These “empty” replicates are not included in computing the coverage rates. As a conditional model, Selective Inference only computes the confidence intervals for the selected predictors.

**Figure A1:**
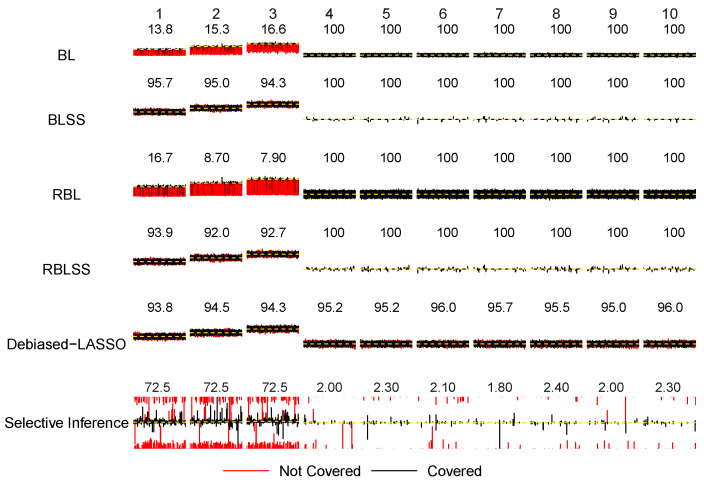
Confidence and credible intervals and their coverage probabilities under a 95% nominal level for the first 10 predictors, where the first 3 are true nonzero coefficients. Data are simulated under independent covariance (n,p)=(100,501) and N(0,1) error across 1000 replicates.

**Figure A2:**
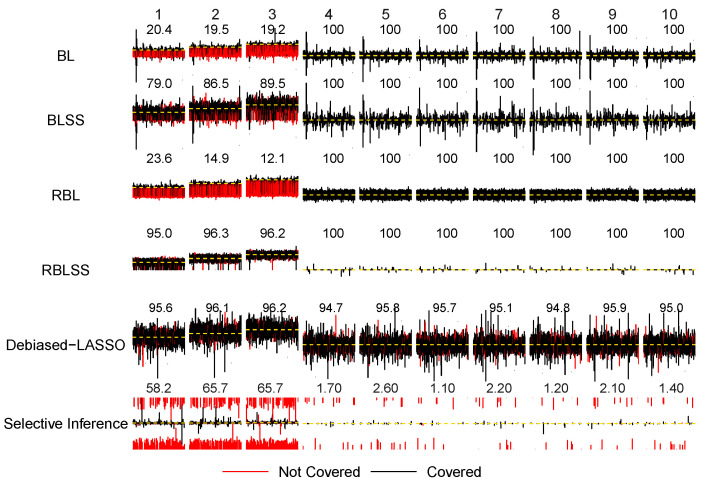
Confidence and credible intervals and their coverage probabilities under a 95% nominal level for the first 10 predictors, where the first 3 are true nonzero coefficients. Data are simulated under independent covariance (n,p)=(100,501) and *t*(2) error across 1000 replicates.

From Figure 2 and Figure 3, we can quickly identify the advantage in terms of inference by adopting robust Bayesian analysis while incorporating exact sparsity. RBLSS yielded satisfactory coverage rates with much narrower credible intervals, especially under the *t*(2) error. Besides, we can see that although Debiased-LASSO can maintain good coverage probability for both the true and false covariates, the confidence intervals are much wider compared with those of RBLSS, especially under the *t*(2) error. Furthermore, the super-efficient phenomenon for all Bayesian approaches on noisy predictors is evident from both Figure 2 and Figure 3. In addition, we have conducted simulations when predictors are generated independently from multivariate normal distribution with diagonal covariance matrix. In the Appendix A, Table A1 shows estimation, identification and inference results. The coverage probabilities are visualized in Figure A1 and Figure A2 for N(0,1) and *t*(2) errors, respectively. Similar conclusions can be reached under this setting.

## 4. Examples

We have provided a selected list of published studies which have developed high-dimensional inference procedures for analyzing genomics data in Table 3. Among them, Wang et al. (2012) [160] has proposed the penalized generalized estimating equation (PGEE) to conduct variable selection with high-dimensional longitudinal data, which typically has strong intra-cluster correlations. Numerical experiments suggest that the confidence intervals obtained based on asymptotic normality of the PGEE estimator can maintain correct coverage probabilities under the nominal level. Inferential tools on sparse longitudinal data have rarely been reported under the methods in Section 2.1.2, Section 2.1.3, and Section 2.1.4. In particular, Kuchibhotla et al. (2022) [24] has pointed out that data splitting based an inference procedure may not work well for a broad array of dependent data. Besides, published studies of [183,185] have developed uncertainty quantification measures under binary outcomes with frequentist and Bayesian methods, respectively.

The tension between prediction and inference discussed in the Introduction has been noted in [6], among other works. Breheny (2019) [6] has shown that in the analysis of TCGA breast cancer data, the tuning parameter corresponding to the smallest CV error has yielded a set of selected gene expressions with a marginal FDR larger than 0.9, suggesting that while this tuning is useful in terms of prediction, one should remain uncertain about whether the selected features are genuinely associated with the disease phenotype. On the other hand, Meinshausen and Bühlmann (2010) [9] has demonstrated that relevant FDR inference measures can facilitate the reproducibility of findings identified by graphical LASSO.

Zhou et al. (2023) [20] is among the first to quantify the uncertainty of nonparametric curve estimates under sparse quantile-varying coefficient models. Although inference measures with non-robust regularized varying coefficient models have been established and validated in [186], similar results are not available for robust VC models in the presence of heavy-tailed distributions and outliers. This aligns with our observations regarding the scarcity of research on robust inferences under sparse models and again reveals the importance of developing robust Bayesian inference procedures for analyzing cancer genomics data.

## 5. Discussion

In this article, we have performed a systematic overview of high-dimensional inference methods from both the frequentist and Bayesian frameworks. We have assessed the estimation, identification and particularly statistical inference performances of six existing shrinkage methods. Our focus is on evaluating the accuracy in inference, which can only be accomplished in simulations when the data-generating model is available. For instance, to compute the coverage probabilities of confidence intervals (or Bayesian credible intervals), the exact true values of the regression parameters must be known in advance. We have not pursued real data analysis, as applications of these methods to cancer genomics data have already been conducted in published studies. Our numerical analysis indicates that fully robust Bayesian variable selection methods can yield honest Bayesian credible intervals, especially in the presence of heavy-tailed errors in the response variable. Evaluations can be further conducted with different signal strengths, noise levels, heavy-tailed errors (both homogeneous and heterogeneous) and quantile levels. Our analysis has shown a promising approach to complementing the lack of robust inferences for frequentist sparse models.

Although the importance of software development for high-dimensional models has been increasingly recognized, only a few packages performing statistical inferences are available for frequentist regularization methods. This is partially due to the fact that estimation and inference of penalization methods are decoupled, as discussed in the Introduction. Therefore, a software package designed to fit sparse models typically provides only estimation and prediction results. It does not yield uncertainty quantification measures unless inference tools are specifically included in the package. For example, Wu and Liu (2009) [96] has established the oracle properties of SCAD and adaptive LASSO-regularized quantile regressions which can be adopted to compute confidence intervals. However, popular R packages for (frequentist) quantile regression have only implemented model-fitting procedures without inferential ones [187,188]. In addition, during the revision of this article, it came to our attention that Han et al. (2022) [101] had assessed finite-sample performance in terms of coverage probabilities for post-selection inference with penalized Huber loss regression. Nevertheless, the R package mentioned in [101] was developed earlier, and is limited to model fitting. It does not yet include an inference procedure.

On the other hand, (robust) Bayesian inference is “off the shelf” because posterior estimation and inference can be conducted simultaneously. Even though packages implementing fully Bayesian methods may not emphasize inferential tools, uncertainty quantification can be routinely performed using posterior samples generated from MCMC. Therefore, Bayesian analysis should be included as a baseline method in cancer research, especially when the inference measures of other approaches are not available. In this study, we have focused on cancer genomics data. We conjecture that similar analysis can be performed for other types data [189].

## Figures and Tables

**Table 1:** A selected list of reviews that have focused on or included high-dimensional inferences.

Reference	Type	Description	Inference Procedure	Numerical Study
Dezeure et al. (2015) [21]	Theory and frequentist	Provided a selective surveys on high-dimensional inference for frequentist regularization methods, which focuses on sample splitting, regularized projection and bias correction. R package hdi has also been introduced.	Confidence intervals and *p*-values in both linear and generalized linear models. Multiple testing correction included.	Both simulation and a case study on riboflavin data.
Fan and Lv (2010) [38]	Theory and frequentist	Systematically overviewed the theory, methods and application in high-dimensional variable selection. Topics on oracle property and ultra-high dimensional variable selection are included.	Discussed the oracle properties in both the classical and ultra-high dimensional setting.	No.
Cai and Sun (2017) [23]	Theory and frequentist	Surveyed recently developed large scale multiple testing with FDR control and examined efficient procedures to handle hierarchical, grouping and depedent structure.	Multiple testing with FDR control.	No.
Kuchibhotla et al. (2022) [24]	Theory and frequentist	Reviewed three categories of inference methods after variable selection: sample splitting, simultaneous inference and conditional selective inference.	Confidence intervals and *p*-values.	No simulation. Case studies on Boston Housing data.
Chernozhukov et al. (2023) [25]	Theory and frequentist	Reviewed recent development on high-dimensional bootstrap including high-dimensional central limit theorems, multiplier and empirical bootstrap and applications.	Confidence intervals and *p*-values.	Provided R codes to compute different versions of *p*-value on hedge fund data (*n* = 50, *p* = 2000).
Huang et al. (2022) [26]	Theory and frequentist	Surveyed statistical tests for high-dimensional mean problems, with a focus on testing two-sample means for differentially expressed gene expression analysis.	Power of tests and control on type 1 error.	Simulation.
Zhang et al. (2022) [27]	Theory and frequentist	A theoretical review on post selection inferences under linear models.	Examined the confidence intervals and coverage probabilities.	Simulation.
Heinze et al. (2018) [28]	Applied (for practicing statisticians) and frequentist	Focus on surveying variable selection methods for low-dimensional problems including backward/forward/stepwise/best subset selection and LASSO.	Inferences were not explicitly examined. Discussed model stability, resampling and bootstrap.	No simulation. Case study on body-fat data (*n* = 251, *p* = 13).
Bühlmann et al. (2014) [29]	Applied and frequentist	Reviewed uncertainty quantification using type 1 error and *p*-values on high-dimensional linear models (including generalized linear models and mixed models), graphical models, and causal inferences.	FDR and *p*-values.	No simulation. A case study on Riboflavin data with *n* = 71 and *p* = 4088.
Benjamini (2009) [10]	Applied and frequentist	Summarized the current success and future trend in inferences with FDR and multiple comparisons.	Discussed False discovery rates (FDR) and Family wise error rates (FWER).	No.
Farcomeni (2008) [30]	Applied and frequentist	Reviewed multiple hypothesis testing with control on different error measures related to FDR and its variants.	Assessed validity of controlling a variety of FDR related error measures in multiple hypothesis testing.	Simulation and two case studies on clinical trials with multiple endpoints and DNA microarrays.
O’hara and Sillanpöö (2009) [3]	Theory and Bayesian	Reviewed major categories of Bayesian variable selection methods, including indicator model selection, adaptive shrinkage, and stochastic search variable selection.	Posterior distributions of regression coefficients and posterior inclusion probabilities.	Both simulation and real data.
Lu and Lou (2022) [31]	Applied and Bayesian	Surveyed Bayesian variable selection under a variety shrinkage priors and conducted comprehensive comparative study.	Coverage probability on prediction assessed on real data.	Simulation and a case study on body-fat data (*n* = 251, *p* = 13).
Fridley (2009) [32]	Applied and Bayesian	Reviewed and compared Bayesian variable and model selection in genetic associations.	Posterior inclusion probability and credible intervals.	Both simulation and case studies on 17 SNPs genotyped from two genes.
Muller et al. (2007) [33]	Theory and Bayesian	Reviewed and compared Bayesian approaches to multiple testing.	Bayesian FDR and its variants.	Real data on DNA microarray studies.
Bhadra et al. (2019) [34]	Theory and Bayesian	Surveyed two major types of variable selection methods, LASSO and Horseshoe, in high-dimensional inference, efficiency and scalability.	Examined theoretical optimality in high-dimensional inference.	Used simulated data to check theoretical assumptions.

**Table 2:** Comparison of all methods in terms of estimation, identification and inference (under a nominal level of 95%) for data with AR(1) correlation (n,p) = (100,501) across 1000 replicates.

		Methods					
		BL	BLSS	RBL	RBLSS	Debiased-LASSO	Selective Inference
Error 1 N(0,1)	$L_{1}$ error of $\beta_{1}^{★}$	0.951	0.305	1.713	0.329	0.688	0.497
	Standard deviation	0.213	0.146	0.284	0.159	0.019	0.017
	$L_{1}$ error of $\beta_{2}^{★}$	10.782	0.312	14.739	0.576	0.061	0.712
	Standard deviation	0.777	0.333	0.818	0.285	0.021	0.085
	TP	2.961	3.000	1.941	3.000	3.000	3.000
	Standard deviation	0.194	0.000	0.419	0.000	0.000	0.000
	FP	0.000	0.096	0.000	0.275	1.430	14.996
	Standard deviation	0.000	0.333	0.000	0.560	0.438	1.503
	Coverage of $\beta_{1}^{★}$						
	$\beta_{1}$	0.774	0.942	0.946	0.920	0.913	0.788
	$\beta_{2}$	0.966	0.945	0.990	0.931	0.775	0.790
	$\beta_{3}$	0.740	0.949	0.795	0.932	0.911	0.784
	Average length						
	$\beta_{1}$	0.937	0.472	1.369	0.476	0.483	-
	$\beta_{2}$	1.075	0.526	1.892	0.531	0.482	-
	$\beta_{3}$	1.008	0.471	1.892	0.477	0.481	-
	Coverage of $\beta_{2}^{★}$	0.994	0.994	0.996	0.994	0.956	0.015
	Average length	0.414	0.006	0.829	0.010	0.482	-
Error 2 *t*(2)	$L_{1}$ error of $\beta_{1}^{★}$	1.602	1.353	2.033	0.486	2.222	1.243
	Standard deviation	0.784	0.941	0.451	0.331	0.103	0.065
	$L_{1}$ error of $\beta_{2}^{★}$	25.542	4.911	23.405	0.383	0.025	1.416
	Standard deviation	0.063	14.225	4.082	0.225	0.017	0.176
	TP	2.200	2.437	1.350	2.948	2.409	2.856
	Standard deviation	0.784	0.612	0.720	0.222	0.093	0.047
	FP	0.004	10.376	0.000	0.042	0.329	13.183
	Standard deviation	0.063	62.511	0.000	0.186	0.212	1.357
	Coverage of $\beta_{1}^{★}$						
	$\beta_{1}$	0.751	0.745	0.906	0.954	0.910	0.722
	$\beta_{2}$	0.898	0.843	0.949	0.960	0.755	0.788
	$\beta_{3}$	0.636	0.906	0.665	0.964	0.901	0.794
	Average length						
	$\beta_{1}$	1.219	1.143	1.448	0.833	1.418	-
	$\beta_{2}$	1.601	1.555	1.971	0.856	1.409	-
	$\beta_{3}$	1.564	1.348	2.004	0.728	1.412	-
	Coverage of $\beta_{2}^{★}$	0.996	0.996	0.997	0.995	0.955	0.015
	Average length	0.704	0.120	0.953	0.008	1.414	-

**Table 3:** A selected list of case studies that have developed high-dimensional inferences in genomics studies.

Reference	Model	Inferences and Software	Case Study
Wang et al. (2012) [160]	Penalized Generalized Estimating Equation (PGEE) for longitudinal data	Confidence interval based on asymptotic property of Oracle estimator. R package *PGEE* (ver 1.5).	Yeast cell-cycle gene expression (GE) data. *n* = 297, *p* = 96. Y: log transformed time varying GE; X: matching score of binding probability
Breheny (2019) [6]	Penalized regression (LASSO, SCAD and MCP)	Marginal FDR for penalized regression. R package *ncvreg* (ver 3.14.3).	(1) TCGA breast cancer data *n* = 536, *p* = 17,322. Y: BRCA1 expression, X:GE; (2) Genetic association study of cardiac fibrosis *n* = 313, *p* = 66,0496 Y: ratio of cardiomyocytes to fibroblasts in the heart tissue on log scale. X: SNP
Xia et al. (2023) [185]	De-biased LASSO in generalized linear models.	Confidence intervals based on refined de-biasing estimating approach.	Boston lung cancer data. Y: binary with 723 controls and 651 cases (*n* = 1374) X: 103 SNPs and 4 clinical covariates
Meinshausen and Bühlmann (2010) [9]	Graphical LASSO and LASSO.	Error control on expected number of falsely selected edges from the graph.	Riboavin (vitamin gene-expression) data with *n* = 115 subjects and *p* = 160 GEs. No phenotype.
Zhang et al. (2014) [183]	Generalized hierarchical structured (bi-level) Bayesian variable selection	Bayesian credible intervals and FDR.	Breast cancer study. Y: binary. Case: 184 TNBC subtype. Control: 787 other and unclassified subtypes. (*n* = 971) X: 167, 574 probes for copy number measurements
Zhou et al. (2023) [20]	Bayesian regularized quantile varying coefficient model.	Bayesian credible intervals on non-linear gene-environment interaction effects. R package *pqrBayes* (ver 1.0.2).	Type 2-diabetes data with SNP measurements. *n* = 1716 Y: BMI; X: 53,408 SNPs

## Data Availability

No new data were created. Simulated data were generated using R codes.

## Abbreviations

The following abbreviations are used in this manuscript:

ADMM	Alternating direction method of multipliers
BL	Bayesian LASSO
BLSS	Bayesian LASSO with spike-and-slab prior
BMI	Body mass index
CI	Confidence interval
CV	Cross-validation
FDR	False discovery rate
GE	Gene expression
GWAS	Genome-wide association study
LAD	Least absolute deviation
LASSO	Least absolute shrinkage and selection operator
MCMC	Markov chain Monte Carlo
MCP	Minimax concave penalty
MPM	Median probability model
PGEE	Penalized generalized estimating
RBL	Robust Bayesian LASSO
RBLSS	Robust Bayesian LASSO with spike-and-slab prior
SCAD	Smoothly clipped absolute deviation
SNP	Single-nucleotide polymorphism

## Appendix A

### Appendix A.1

**Table A1:** Comparison of all methods in terms of estimation, identification and inference (under a nominal level of 95%) for data with independent covariance structure (n,p) = (100,501) across 1000 replicates.

		Methods					
		BL	BLSS	RBL	RBLSS	Debiased-LASSO	Selective Inference
Error 1 N(0,1)	$L_{1}$ error of $\beta_{1}^{★}$	1.773	0.250	2.898	0.274	1.038	0.718
	Standard deviation	0.312	0.113	0.299	0.121	0.026	0.023
	$L_{1}$ error of $\beta_{2}^{★}$	11.891	0.331	16.553	0.601	0.128	1.021
	Standard deviation	0.797	0.176	0.681	0.291	0.027	0.100
	TP	2.696	3.000	0.906	3.000	3.000	3.000
	Standard deviation	0.460	0.000	0.549	0.000	0.000	0.000
	FP	0.000	0.112	0.000	0.341	3.057	21.533
	Standard deviation	0.000	0.357	0.000	0.647	0.547	1.722
	Coverage of $\beta_{1}^{★}$						
	$\beta_{1}$	0.138	0.957	0.167	0.939	0.938	0.725
	$\beta_{2}$	0.153	0.950	0.087	0.920	0.945	0.725
	$\beta_{3}$	0.166	0.943	0.079	0.927	0.943	0.725
	Average length						
	$\beta_{1}$	0.789	0.412	1.030	0.416	0.458	-
	$\beta_{2}$	0.901	0.411	1.309	0.413	0.456	-
	$\beta_{3}$	0.908	0.410	1.607	0.412	0.457	-
	Coverage of $\beta_{2}^{★}$	0.995	0.994	0.998	0.994	0.957	0.020
	Average length	0.405	0.006	0.814	0.010	0.456	-
Error 2 *t*(2)	$L_{1}$ error of $\beta_{1}^{★}$	2.549	1.096	3.125	0.376	3.049	1.730
	Standard deviation	0.551	0.881	0.372	0.285	0.103	0.081
	$L_{1}$ error of $\beta_{2}^{★}$	27.124	8.645	23.650	0.390	0.067	2.099
	Standard deviation	39.807	40.405	3.622	0.229	0.021	0.213
	TP	1.581	2.611	0.474	2.972	1.996	2.731
	Standard deviation	0.793	0.610	0.558	0.208	0.117	0.066
	FP	0.007	15.999	0.000	0.039	0.849	19.204
	Standard deviation	0.083	78.785	0.000	0.223	0.247	1.632
	Coverage of $\beta_{1}^{★}$						
	$\beta_{1}$	0.204	0.790	0.236	0.950	0.956	0.582
	$\beta_{2}$	0.195	0.865	0.149	0.963	0.961	0.657
	$\beta_{3}$	0.192	0.895	0.121	0.962	0.962	0.657
	Average length						
	$\beta_{1}$	0.969	1.073	1.106	0.666	1.282	-
	$\beta_{2}$	1.218	1.127	1.636	0.629	1.281	-
	$\beta_{3}$	1.374	1.152	1.653	0.631	1.276	-
	Coverage of $\beta_{2}^{★}$	0.997	0.996	0.999	0.994	0.954	0.018
	Average length	0.703	0.208	0.923	0.007	1.282	-
